# Association between Epstein Barr virus and Oral Lichen Planus clinical phenotypes and p53 expression

**DOI:** 10.1038/s41598-025-12095-3

**Published:** 2025-07-24

**Authors:** Suhail H. Al-Amad, Raed O. AbuOdeh, Vidhya Anish Nair, Wael M. Abdel-Rahman

**Affiliations:** 1 Department of Oral and Craniofacial Health Sciences, College of Dental Medicine, University of Sharjah, Sharjah, United Arab Emirates; 2 Department of Medical Laboratory Sciences, College of Health Sciences, University of Sharjah, Sharjah, United Arab Emirates; 3 Research Institute of Medical and Health Sciences, University of Sharjah, Sharjah, United Arab Emirates

**Keywords:** Epstein Barr virus, Oral Lichen Planus, Erosive lichen planus, Malignant potential, p53, In situ hybridization, Plasma cells, Oral diseases, Skin diseases

## Abstract

To investigate the association between Epstein Barr virus (EBV) and Oral Lichen Planus (OLP) in both of its clinical phenotypes (erosive and non-erosive), 33 OLP cases divided into erosive and non-erosive phenotypes, and 26 non-OLP cases were evaluated for the presence of EBV using In Situ Hybridization. Immunohistochemistry was used to assess expressions of CD3, CD20, CD138 and p53 in both OLP clinical phenotypes. EBV was detected in 11 (33%) of the OLP cases and none of the non-OLP cases (*p* = 0.002). CD3 and CD20 were both over-expressed in all OLP cases, however CD138 was significantly over-expressed in the erosive OLP phenotype by comparison to the non-erosive one (*p* = 0.003), suggesting a possible role of plasma cell in erosive OLP. There was no association between EBV and CD-138, nor with erosive OLP. Interestingly however, EBV had an association with p53 expression among OLP cases (*p* = 0.038), inferring a role of EBV in possible neoplastic changes in OLP. There appears to be a potential role of EBV in causing at least some OLP cases, regardless of whether OLP is erosive or not. EBV might have an etiological role in causing some OLP cases. This could explain why some OLP cases are recalcitrant to corticosteroids treatment. Clinical trials are needed to establish whether EBV-infected OLP cases respond to antiviral therapy.

## Introduction

Oral Lichen Planus (OLP) is an oral mucosal disease with a worldwide pooled prevalence rate of 1.01%^1,2^. Apart from the mouth, lichen planus can be seen in the skin, genitalia and fingernails^3,4^, but the oral form of lichen planus is the one commonly associated with symptoms.

OLP presents in various clinical forms that include reticular, plaque-like, atrophic, erosive, or a combination of two or more of those clinical forms^5^. Although distinction between the various clinical subtypes carries little clinical significance, pain symptoms are often distinct between two broad clinical phenotypes: erosive and non-erosive OLP, with the former being up to 3 times more painful, and recalcitrant to treatment than the latter^5–9^.

Another important distinction between the erosive and non-erosive OLP phenotypes lies in the former’s greater expression of p53 protein^11–13^ and greater potential for malignant transformation^1,10,11^. This clinical and molecular characterization of the erosive OLP, by comparison to non-erosive OLP, raises the question whether this clinical phenotype represents a distinct disease entity with distinct etiopathogenesis.

The exact etiology of OLP remains unknown, but several studies reported associations between the occurrence and severity of OLP and local factors such as metal-based dental restorations and hypersensitivities to foods^14,15^. Oral microbiota has recently been found to differ between OLP and non-OLP patients^16^. Diversity in microbial presence between OLP and non-OLP patient was reported for *Candida albicans*^17^, Hepatitis C virus^18,19^, human papillomavirus^20^, and Epstein–Barr virus (EBV)^7,21,22^, suggesting a possible role of microorganisms in the etiology of OLP.

EBV is a herpesvirus that is highly contagious. More than 90% of adults have antibodies against EBV, more than half of whom demonstrate detectable EBV-DNA^23^. The prevalence increases with age^23,24^, and the mode of viral transmission is often by saliva, with possible transmission through blood transfusion and organ transplantation^23^.

Following EBV’s primary infection (known as infectious mononucleosis), the virus survives indefinitely in the host’s B cells, generating a condition of latent infection with occasional reactivations that manifest in a diversity of neoplastic and chronic inflammatory diseases in various body organs, including oral hairy leukoplakia, Burkitt’s lymphoma, Hodgkin’s lymphoma, nasopharyngeal carcinoma, oral squamous cell carcinoma, gastric carcinoma^25–27^, systemic lupus erythematosus^28^ and multiple sclerosis^29^…among others.

Possible associations between EBV and OLP have previously been investigated with results favoring an association^21,22^ and others refuting it^30,31^. This diversity in conclusions can be attributed to variations in OLP diagnostic criteria, methodological heterogeneity that utilized various techniques to detect EBV such as In Situ Hybridization, immunohistochemistry, polymerase chain reaction, and electron microscopy^21,22,32^, or the possibility that OLP represents more than one disease entity (for example, erosive and non-erosive types) one of which being caused by EBV.

An etiological distinction between erosive and non-erosive OLP in terms of oral microbiota has been reported^16^, suggesting the possibility that erosive OLP represents a distinct disease entity with a distinct etiopathogenesis that might be related to viruses.

The conflicting evidence in the literature regarding associations between EBV and OLP warrants further investigations with emphasis on OLP’s clinical phenotype being erosive or non-erosive. Accordingly, the current study aimed at answering three questions; firstly, whether there is an association between EBV and OLP; secondly, whether EBV -if found in OLP- has an association with the OLP’s erosive clinical phenotype; and thirdly, whether EBV has an association with p53 expression among OLP lesions.

## Materials and methods

### Ethics approval

This study was reviewed and approved by both the University of Sharjah Research Ethics Committee (approval number REC-19-11-25-01) and the Hospital Ethics and Research Committee of the University Hospital Sharjah (approval number UHS-HERC-114-12102022). Both ethics committees have granted waiver of informed consent for this study since it is a retrospective study on archival material. All methods were carried out in accordance with the Declaration of Helsinki 1964 and its later amendments.

### Sample

To assess significant difference in prevalence of EBV between OLP cases and non-OLP controls, we calculated the sample size based on the reported prevalence of EBV in OLP as 74%^22^ and in normal oral mucosal tissues as 27%^33^. Using a two-sided test with a significance level of 0.05 and a power of 90%, the minimum required sample size was calculated to be 19 for each group.

Formalin-fixed paraffin-embedded oral biopsies (n = 61), that have been obtained between 2010 and 2023, were retrieved from the pathology archives of the University Dental Hospital Sharjah and the University Hospital Sharjah. Blocks were retrieved for analysis if the biopsy specimen was diagnosed as OLP (considered cases) or as fibroepithelial, reactive epithelial hyperplasia, frictional keratosis (considered controls). Tissue blocks with no or inadequate viable tissue upon sectioning were excluded (n = 6).

**Fig. 1:**
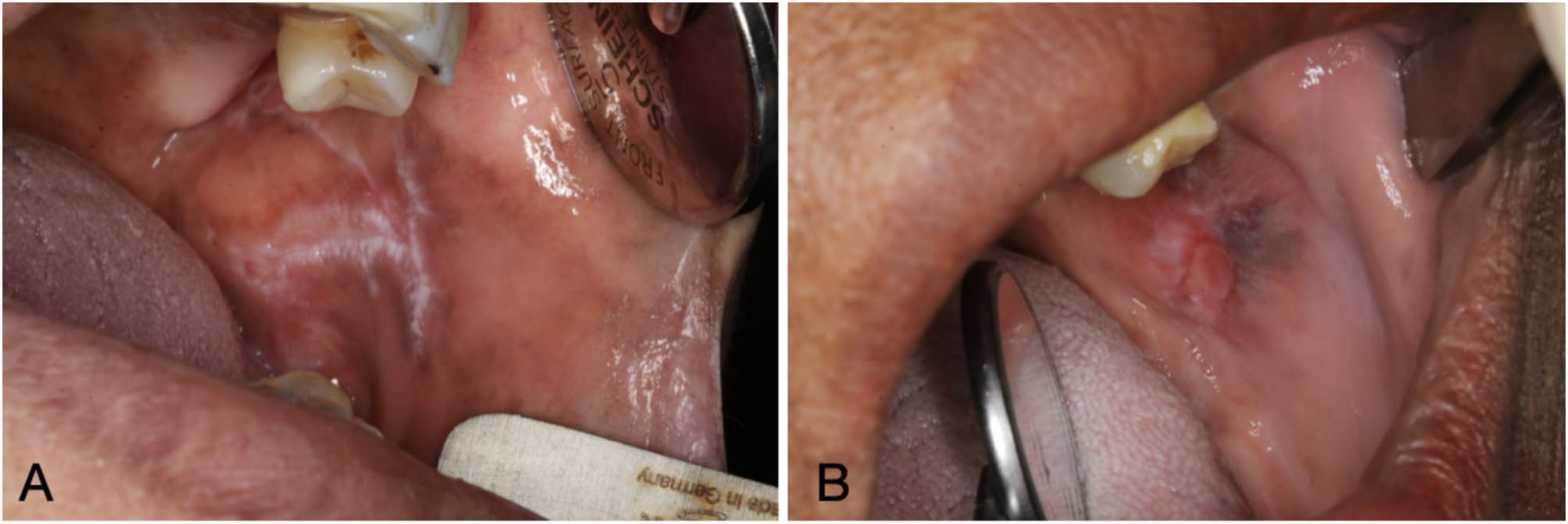
Clinical images of Oral Lichen Planus. (**A**): reticular non-erosive type. (**B**): erosive type.

OLP cases were further classified into erosive and non-erosive based on high resolution clinical photographs of the OLP lesions. Photographs were taken at the time of biopsy, using Canon 70D digital SLR camera supported by 100 mm macrolens and ring flash (Canon Inc. Japan). Classification was performed by an Oral Medicine consultant (SA (author)) based on the criteria adapted from Escudier et al.^5^ whereby OLP cases were considered erosive if lesions showed marked erythema, and/or eroded/ulcerated mucosa with yellow fibrin slough. OLP cases were considered non-erosive if the lesions showed plaque-like or reticular keratotic changes, with or without mild erythema^5^ (Fig. 1). At the time of this classification, assessor (SA (author)) was blinded to the results of the EBV, CD3, CD20, CD138 and p53. All biopsies (OLP cases and controls) had been obtained for clinical purposes, and the diagnosis of OLP or otherwise was made by licensed consultants in Anatomical Pathology or Oral Pathology prior to this study and further confirmed by (WMA-R (Pathologist and co-author)) prior to immuno-histochemistry and in situ hybridization tests. The pathologist who assessed those histological samples was blinded to OLP’s clinical phenotype.

### Immunohistochemical analysis

Biopsy specimens were sectioned into slices with a thickness of 4 µm and mounted on positive charged slides. After deparaffinization and rehydration, the antigen retrieval was performed with EDTA pH 8.0 for 5 min at 750 W followed by 5 min at 450 W in a microwave oven. Sections were washed with Tris-buffered saline (TBS). The staining area was manually marked with a Dako pen (Agilent Technologies, Santa Clara, CA, USA) before the staining steps. The detection reagents were from the mouse and rabbit specific HRP/DAB (ABC) detection IHC kit (Abcam, Cambridge, UK). The kit has a complete set of reagents including peroxidase- and protein-blocking reagents, biotinylated secondary antibody, streptavidin-HRP, and the DAB substrate. Subsequent steps were performed according to the manufacturer’s protocol. The primary antibodies were CD20 for B cells (clone L26; Dako/Agilent), CD3 for T cells (polyclonal rabbit; Dako/Agilent), CD138 for plasma cells (clone MI15; Dako/Agilent), and p53 protein (clone DO-7; Dako/ Agilent). Sections were then incubated with primary antibody overnight at + 4 °C for all antibodies, then rinsed with TBS. Sections were counterstained with Mayer’s hematoxylin.

Analysis of immunohistochemistry was performed based on the protocol reported by Raybaud et al. with modifications^22^. The number of positively stained cells in 5 fields (× 100 power) were counted in each section where there was enough tissue. If the tissue available for counting was less than 5 fields, the total number obtained from the fields available was multiplied by a factor of 5. The final score for each of CD20, CD3, CD138 and p53 was recorded as a continuous variable, and further categorized into dichotomous categories of negative and positive, where positive category was considered if the score was > 0. p53 immune reactivity was considered positive if seen in either epithelial cells and/or inflammatory cells.

### EBV detection

EBV-infected cells (EBV + cells) were detected according to EBER-ISH performed with the ZytoFast EBV-CISH Probes-Digoxigenin-labelled, cat number T-1114-400 and detection was by ZytoFast PLUS CISH Implementation Kit HRP-DAB, cat number T-1063-40, from ZytoVision GmbH, Bremerhaven Germany as per the manufacturer’s protocols. A positive tissue control was included in each run.

Interpretation of EBV results was performed using a protocol modified from that reported by Raybaud et al.^22^. The level of infiltration of EBV-positive cells was evaluated by counting the number of positive cells in 5 × 100 power fields in each section where there was enough tissue. . If the tissue available for counting was less than 5 fields, the total number obtained from the fields available was multiplied by a factor of 5. The final score was recorded as a continuous variable and further categorized into dichotomous categories of negative and positive. For simplicity, a positive category was defined as a score greater than 0.

### Statistical analysis

Association between EBV positivity and patients’ sex (male or female), OLP diagnosis (cases, controls), and CD3, CD20, CD138 and p53 expression (positive or negative for each) as categorical variables was done using Chi-square test. Association between EBV positivity and patient age was assessed using Independent Samples *t*-test. Mean ranks of EBV scores and the scores of CD3, CD20, CD138 and p53 expressions as continuous variables among the erosive and non-erosive OLP was assessed using Mann Whitney U tests. Spearman correlation test was used to assess the association between the scores of CD3, CD20, CD138 and p53 expression among OLP cases. Statistical analysis was performed using SPSS (IBM Corp. Released 2021. IBM SPSS Statistics for Windows, Version 28.0. Armonk, NY: IBM Corp). *p*-value was considered significant if =  < 0.05 two-tailed.

## Results

Among the 55 samples studied, 33 (60%) were of OLP lesions (cases) and 22 (40%) were of non-OLP lesions (controls). The mean age of OLP cases and non-OLP controls was 46.5 (SD = 11.8) and 45.6 (SD = 13.4) years, respectively. Male-to-female ratio for the OLP and non-OLP cases was 2.3:1 and 1.4:1, respectively. There was no statistically significant difference between cases and controls in terms of patients’ age and sex (Mann Whiteney U-test = 343.5 (p = 0.74)) and (X^2^ = 0.27 (p = 0.6)), respectively.

**Fig. 2:**
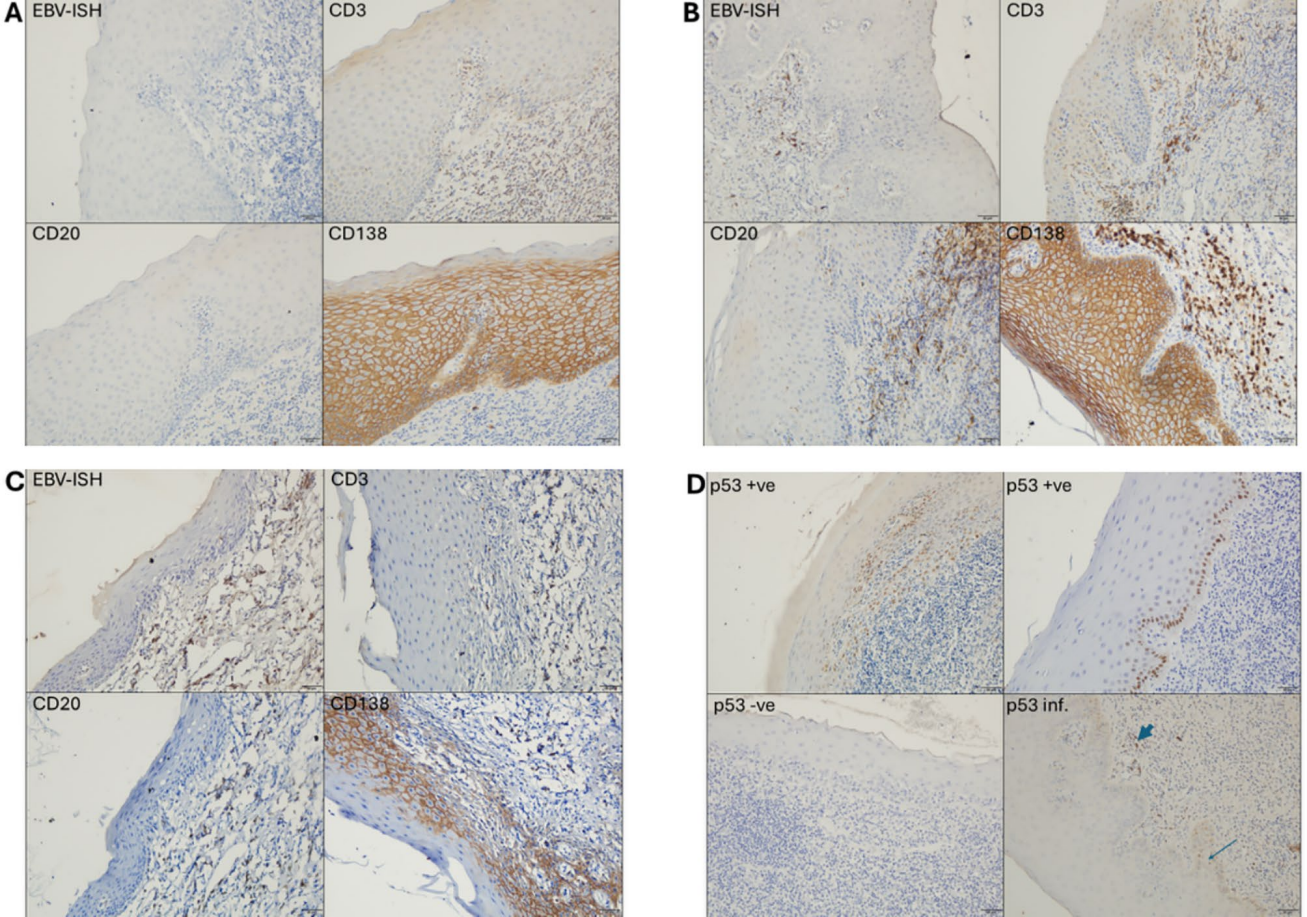
Serial sections prepared from formalin-fixed, paraffin-embedded biopsies taken from OLP cases and non-OLP controls (not shown), were stained for EBV using ISH. Immunohistochemistry was performed to detect T cells (CD3), B cells (CD20), plasma cells (CD138), and p53. (A), representative images from EBV negative case, (B) and (C) are representative images from EBV positive cases, CD138 positivity in epithelial cells is a known non-specific reaction which served as positive staining control. (D) different patterns of p53 immunohistochemistry expression; noting that “p53 inf.” was a rare pattern where we noticed p53 positivity in inflammatory cells (thick arrow) rather than epithelial cells (thin arrow).

Among the entire 55 samples, EBV was detected in 11 (20%) of the sample, all of which were OLP cases (*p* = 0.002) (Fig. 2). No significant association was found between EBV-positive samples and patient sex or age (Table 1).

**Table 1:** Association between EBV positivity and OLP lesions, patients’ sex and patients’ age.

Variable		EBV detection		*p*-value*
		Negative N (%)	Positive^∧^ N (%)	
Diagnosis	Non-OLP	22 (100%)	0 (0%)	0.002
	OLP	22 (66.7%)	11 (33.3%)	
Patient sex	Male	27 (75.0%)	9 (25.0%)	0.20
	Female	17 (89.5%)	2 (10.5%)	

Variable	EBV detection		*p*-value**
	Negative Mean (SD)	Positive Mean (SD)	
Patient age	45.6 (11.4)	46.5 (11.8)	0.80

*Based on Chi-square test.

**Based on Independent Samples t-test.

^∧^ Positivity was defined as values greater than zero.

### ISH, in situ hybridization; EBV, Epstein Barr virus. Magnification, × 200; chromogen, DAB; counterstain, Mayer’s hematoxylin.

Among the OLP cases (n = 33), CD3 was detected in all samples indicating a prominent presence of T-lymphocytes. CD20 and CD138 were positive in the majority of samples, indicating a strong presence of B-lymphocytes and plasma cells, respectively. p53 -on the other hand- was positive in 77% of biopsies (Table 2).

**Table 2:** Expression of CD138, CD20, CD3 and p53 among OLP cases (n = 33).

Variable	Positive* N (%)	Negative N (%)
CD138	27 (81.8%)	6 (18.2%)
CD20	30 (93.8%)	2 (6.3%)
CD3	33 (100%)	0 (0%)
p53	24 (77.4%)	7 (22.6%)

* Positivity was defined as values greater than zero.

EBV detection did not correlate with the expressions of CD3, CD20, CD138 or p53. A significant positive correlation was seen between CD20 and both CD138 and CD3 (Table 3).

**Table 3:** Correlation between EBV expression scores and that of CD3, CD20, CD138 and p53 among the OLP cases (n = 33).

	CD138 *r*_*s*_ (*p*-value)*	CD20 *r*_*s*_ (*p*-value)*	CD3 *r*_*s*_ (*p*-value)*	p53 *r*_*s*_ (*p*-value)*
EBV	0.176 (0.256)	− 0.043 (0.787)	− 0.058 (0.716)	0.210 (0.256)
CD138	–	0.450 (0.003)	0.206 (0.191)	− 0.123 (0.509)
CD20	–	–	0.427 (0.005)	-0.184 (0.330)
CD3	–	–	–	-0.124 (0.507)

*Based on Spearman correlation test.

Among OLP cases, a significant association was seen between the EBV detection and p53 expression. EBV detection was also examined for association with the expression of CD20 and CD138, however this association was not statistically significant (Table 4).

**Table 4:** Association between EBV detection (detected/not detected) and the expression of CD20 and CD138 (expressed or not expressed) among the OLP cases (n = 33).

		EBV detection**		*p*-value*
		Not detected N (%)	Detected N (%)	
CD138	Not expressed	6 (100%)	0 (0%)	0.019
	Expressed**	16 (59.3%)	11 (40.7%)	
CD20	Not expressed	2 (100%)	0 (0%)	0.325
	Expressed**	20 (66.7%)	10 (33.3%)	
p53	Not expressed	7 (100%)	0 (0%)	0.038
	Expressed	14 (58.3%)	10 (41.7%)	

*Based on Chi-square test.

**Positivity was defined as values greater than 0.

(Note: All OLP cases were positive for CD3, the result of cross-tabulation test is therefore not shown in the table above).

Six (20.7%) of the OLP cases were classified as erosive and 23 (79.3%) were classified as non-erosive (Fig. 1). Erosive OLP exhibited higher mean rank values of EBV, CD20 and CD138; however, this association was only statistically significant for CD138 (Table 5).

**Table 5:** Comparison between the mean ranks of EBV, CD138, CD20 and CD3 detection scores among erosive and non-erosive OLP (N = 29)**

	Non-erosive OLP / Erosive OLP	Number of Positive cases	Number of Negative cases	N (mean rank)	U-test	p-value*
EBV	Non-erosive OLP	8	15	23 (14.83)	73.0	0.800
	Erosive OLP	2	4	6 (15.67)		
CD138	Non-erosive OLP	18	5	23 (12.63)	123.5	0.003
	Erosive OLP	6	0	6 (24.08)		
CD20	Non-erosive OLP	21	2	22 (13.14)	96.0	0.092
	Erosive OLP	6	0	6 (19.5)		
p53	Non-erosive OLP	20	2	22 (15.57)	42.5	0.184
	Erosive OLP	3	3	6 (10.58)		

*Based on Mann Whitney U test.

** Four cases of OLP were not included because clinical images and/or clinical details needed to classify lesions into erosive or non-erosive phenotypes were not available.

(Note: During processing, the tissue sample of a single non-erosive OLP cases inadvertently detached from the slide, resulting in the failure to perform CD20 and p53 IHC tests).

## Discussion

OLP is an immune-mediated oral mucosal disease that affects 1.01% of the world’s population, with higher prevalence rates in females and individuals over 40 years of age^1,2,22^. The exact aetiology of OLP remains unknown, although several studies have linked it to various factors such as dental materials (particularly amalgam dental restorations), artificial flavors in foods and oral hygiene products, toothpastes containing sodium lauryl sulphate, and several systemic medications^14,15^. Recent studies found associations between OLP and various microorganisms, suggesting a possible role of specific microorganisms in OLP’s etiology^16,17,20^.

Association between OLP and Epstein-Barr Virus (EBV) has previously been studied with conflicting results^7,21,22^. This heterogeneity in results might be attributed to variations in OLP diagnostic criteria or methodological heterogeneity that utilized various techniques to detect EBV such as immunohistochemistry, polymerase chain reaction, and electron microscopy, or In Situ Hybridization ISH^21,22,32^. In our study, EBV genome was visualized via ISH, which is considered an accurate and powerful method with various applications in clinical pathology and research^34,35^ that can detect viral DNA or RNA in small tissue quantities, such as oral biopsy samples used in our study^36^.

EBV is a herpesvirus classified under the genus Lymphocryptovirus or Human Herpesvirus-4. Seroprevalence studies showed a high prevalence rate among adults exceeding 90%, more than half of whom had detectable EBV-DNA^24^. The virus initially infects oral epithelial cells, mostly through saliva^23,37^, then survives indefinitely in the host’s B lymphocytes, generating a condition of latent infection with occasional reactivations^38^.

Prevalence rates of EBV in oral mucosa biopsies ranged from 27% in normal oral mucosa to 72% in oral potentially malignant disorders (including OLP) and oral cancers^33^*.* In our sample, EBV was detected in one-third of OLP samples, while none were found in the controls, suggesting a potential role for the virus in the etiology of at least a subset of OLP cases. This result is similar to that reported by Ashraf et al. and Raybaud et al.^21,22^ who detected EBV in OLP lesions, specifically residing in infiltrating plasma cells. Similarly, our findings showed that all OLP cases that were positive for EBV were also positive for CD138, inferring that EBV existence in the OLP lesions is vehicled by plasma cells.

OLP manifests in different clinical forms and with various degrees of pain intensity. Different scoring systems have been developed to objectively classify the severity of OLP^39^. Most of those scoring systems rely on the three parameters, namely: Pain intensity, OLP’s anatomical extent, and its clinical phenotype. In our study, we divided OLP into two broad clinical phenotypes of erosive and non-erosive, based on classification criteria adapted from Escudier et al.^5^. Our aim was to assess whether the erosive OLP phenotype, by comparison to non-erosive OLP, was the one infected with EBV. Our results did not show an association between the EBV detection and erosive OLP phenotype. Interestingly though, CD138 was expressed in all the six erosive OLP cases, supporting the assumption that erosive OLP is plasma cell-mediated and possibly represents a distinct disease entity and warrants clinical trials assessing the value of therapeutic agents targeting plasma cells.

Although being primarily a T-cell-mediated disease, the presence of plasma cells in OLP’s inflammatory infiltrate is common^40^, and is mainly seen in oral lichenoid lesions (a variant of OLP that is considered a delayed hypersensitivity reaction to an external factor)^41^. The significant association between CD138-positive cells and EBV in our sample suggests that EBV’s presence in the inflammatory infiltrate is by infecting plasma cells. Raybaud et al. showed that EBV-positive plasma cells can produce viral particles within OLP lesions, implying localized amplification of EBV infection^22^. Thus, the association of CD138-positive plasma cells with OLP in our study most likely implies an EBV amplification role, even though we cannot exclude it being a secondary phenomenon due to ulceration and secondary infection.

Erosive OLP is also known to carry greater malignant potential by comparison to non-erosive OLP^1,10^. Given that EBV is a known oncogenic virus linked to various human cancers, including oral squamous cell carcinoma, we aimed to investigate possible association between this virus and early neoplastic changes in OLP, irrespective of its clinical phenotype. Our investigation focused on lesional p53 expression, a well-established marker of early neoplastic changes, including those seen in OLP^11–13,42^. Although our results revealed no association between EBV and the erosive phenotype of OLP, we observed a significant association between EBV detection and p53 expression in all OLP cases (both erosive and non-erosive). This result was based on chi-square test, but not on Spearman’s correlation coefficient. The discrepancy between both tests is probably attributed to both EBV and p53 data being non-monotonic (in their formats as continuous variables). The significant association between both variables in their categorical format suggests that some form of association between them exists, which calls for further research into a possible role of EBV in pro-oncogenic molecular processes mediated by mutations in the tumor suppressor gene *TP53*.

The role of EBV infection in the pathogenesis of OLP can be explained by a few possible molecular mechanisms. The EBV-encoded latent membrane protein 1 (LMP1) is a well-established viral oncoprotein that can activate multiple signaling pathways, including NF-κB, MAPK/PI3K/STAT, and p53 pathways^43^. LMP1 has been shown to induce epithelial cell transformation and modulate inflammatory responses by promoting the secretion of pro-inflammatory cytokines such as IL-1, IL-6, and TNF-α^38^. These cytokines are known to play crucial roles in the pathogenesis of OLP by recruiting and activating T lymphocytes, which are the predominant inflammatory cells in OLP lesions.

Our finding of a significant association between EBV detection and p53 expression in OLP lesions suggests a potential mechanistic link. Recent studies have demonstrated that EBV infection can lead to p53 protein accumulation through various mechanisms^44^. EBV-encoded proteins, particularly EBNA1 and EBNA3C, can interact with and stabilize p53, leading to its accumulation without necessarily activating its transcriptional function^45^. Additionally, EBV infection can induce cellular stress responses, including DNA damage and oxidative stress, which are known triggers for p53 upregulation^43^. The upregulated p53 in OLP lesions may represent an antitumor response, as suggested by Keim-Del Pino et al.^46^, who demonstrated frequent p53 protein upregulation in OLP patients, indicating a potential protective mechanism against malignant transformation.

Furthermore, EBV may contribute to OLP pathogenesis through epigenetic modifications. EBV has been shown to alter host cell gene expression through DNA methylation, histone modifications, and microRNA regulation^38^. These epigenetic changes could affect genes involved in immune regulation, cell cycle control, and apoptosis, potentially contributing to the chronic inflammatory nature of OLP and the occasional malignant transformation observed in some cases.

While our study focused on EBV’s role in OLP, several other viruses have been implicated in OLP pathogenesis, including Human Papilloma Virus (HPV), particularly with high-risk genotypes 16 and 18^47^. This association was stronger in erosive OLP compared to non-erosive forms^48^, suggesting that HPV might contribute to the more severe clinical manifestations of OLP, unlike our findings with EBV, which showed no preferential association with erosive OLP. HSV has also been investigated in OLP pathogenesis, although with more conflicting results^49^. Interestingly, systemic acyclovir as an adjuvant therapy improved clinical progress of OLP^50^.

The mechanisms by which these viruses contribute to OLP pathogenesis appear to differ. While EBV primarily infects B lymphocytes and may modulate immune responses through LMP1 and other viral proteins as discussed earlier, HPV directly infects epithelial cells and can induce cellular transformation through E6 and E7 oncoproteins, which interfere with p53 and retinoblastoma protein functions^48^. HSV, on the other hand, may trigger OLP through molecular mimicry, where viral antigens share structural similarities with self-antigens, potentially leading to autoimmune reactions^51^.

The apparent disconnect between EBV detection and CD20 expression in our study warrants further discussion. While EBV is known to establish latency predominantly in B cells, recent evidence suggests that in oral mucosal environments, EBV may preferentially persist in plasma cells, which are terminally differentiated B cells that have downregulated CD20 expression while upregulating CD138 (syndecan-1)^52^. This phenomenon has been observed in other EBV-associated oral conditions, such as oral hairy leukoplakia, where EBV can be detected in epithelial cells and plasma cells rather than CD20 + B cells^53^.

The association between EBV and CD138-positive plasma cells in our study aligns with findings by Raybaud et al. and Mahdavi et al.^22,40^, who reported that EBV in OLP lesions primarily resides in infiltrating plasma cells. This suggests that in the context of OLP, EBV may preferentially infect or persist in plasma cells rather than CD20-positive B cells, potentially explaining the lack of significant association between EBV and CD20 expression in our results.

Our research revealed a number of interesting outcomes. Firstly, our study showed a significant association between EBV and OLP, suggesting a possible role of the virus in the etiology of OLP, which highlights the need for further studies involving larger sample sizes to substantiate this potential causal relationship. Secondly, our results revealed a significant association between EBV and p53 in OLP lesions, suggesting a potential role for EBV in neoplastic transformation that has been reported in rare cases of OLP.

Despite these findings, our study is limited by the relatively small sample size which restricts the generalizability of our results. Interpretation of results related erosive versus non-erosive OLP cases should be done with caution due to the relatively small number of erosive OLP phenotypes. The fact that we relied on a retrospective evaluation of clinical photographs to determine the erosive nature of OLP carries some degree of subjectivity, which is another limitation of our study. Finally, scoring and counting ISH and IHC expressions was done semi-objectively by a single observer, which is another limitation that could have been minimized by using an automated image analysis. Our cut-off value of > 0 favors sensitivity in that any detectable presence of EBV or CD markers is considered “positive”, which is suitable for clinical interpretability, but carries the risk of false positives due to background signal and potentially compromises specificity.

## Data Availability

The datasets used and/or analysed during the current study is available from the corresponding author on reasonable request.
